# Aneurysm of a Supra-Articular Branch of the Popliteal Artery Mimicking a Soft-Tissue Sarcoma: A Case Report

**DOI:** 10.7759/cureus.111484

**Published:** 2026-06-25

**Authors:** Mokhtar El Mekhtoume, Younes Abdourabbih, Salma Abouchiba, Hajar Ouazzani, Ismail Chaouche, Amal Akammar, Meriem Haloua, Badreddine Alami, Y. Lamrani, Meryem Boubbou, Mustapha Maaroufi, Nizar El Bouardi

**Affiliations:** 1 Department of Radiology, Hassan II University Hospital, Sidi Mohammed Ben Abdellah University, Fez, MAR; 2 Department of Mother and Child Radiology, Hassan II University Hospital, Sidi Mohammed Ben Abdellah University, Fez, MAR

**Keywords:** aneurysm, atherosclerosis, computed tomography angiography, doppler ultrasound, magnetic resonance imaging, popliteal artery aneurysm, popliteal mass, soft-tissue sarcoma mimicker, supra-articular branch, vascular pitfall

## Abstract

We report an unusual case of a partially thrombosed aneurysm arising from the descending genicular artery, a supra-articular collateral branch of the popliteal artery, in an 80-year-old man, whose clinical and radiological features initially mimicked a soft-tissue sarcoma. The patient was referred for image-guided biopsy of a left popliteal mass that had been progressively enlarging over nine months and had become bothersome during walking. Magnetic resonance imaging (MRI) revealed a well-circumscribed, heterogeneous lesion with hypointense areas on T1- and PD-weighted images, initially interpreted as a fibrous component suggestive of a sarcoma. A pre-biopsy Doppler ultrasound subsequently demonstrated intralesional arterial flow, prompting computed tomography angiography (CTA), which confirmed a partially thrombosed aneurysm of the descending genicular artery, arising from the supra-articular segment of the left popliteal trunk. The main popliteal artery itself was of normal caliber. The most likely etiology was degenerative, given the patient’s age, the diffuse atherosclerotic changes seen on imaging, and the absence of any traumatic, iatrogenic, or systemic predisposing factor. The patient subsequently underwent surgical exclusion of both the aneurysm and its feeding artery, with an uneventful postoperative course. This case illustrates the misleading appearance that aneurysms of popliteal artery branches may have on MRI, and emphasizes that any popliteal mass should be evaluated with Doppler ultrasound before any biopsy is considered, to avoid potentially life-threatening hemorrhage.

## Introduction

Popliteal artery aneurysms (PAAs) are the most common peripheral arterial aneurysms, accounting for approximately 70% of lower-limb aneurysms, with a male predominance and an insidious natural course often nicknamed the “silent killer of the leg circulation” [1].

By striking contrast, aneurysms developed from the collateral branches of the popliteal artery are exceedingly rare, with only isolated cases reported worldwide. The collateral branches of the popliteal artery include the genicular arteries (supero-medial, supero-lateral, middle, infero-medial, and infero-lateral) and the descending genicular artery; these are small-caliber vessels that form a rich anastomotic network around the knee joint, contributing to the genicular plexus and providing collateral perfusion to the leg. The few series available concern almost exclusively pseudoaneurysms of these genicular branches of post-traumatic or iatrogenic origin [2,3,4]. True degenerative aneurysms of these collateral branches, in the absence of any aneurysmal dilatation of the main popliteal artery, are particularly uncommon and have been poorly characterized in the radiological literature.

Their clinical and radiological presentation can be highly misleading. When partial mural thrombosis is present, the MRI appearance may simulate a heterogeneous soft-tissue mass and mimic a malignant tumor such as a sarcoma. Failure to recognize this diagnostic pitfall may prompt unnecessary investigations, delay appropriate vascular management, and, most importantly, lead to an inappropriate percutaneous biopsy with the risk of catastrophic hemorrhage [5].

We report the case of an 80-year-old man with a true degenerative aneurysm of the descending genicular artery, a supra-articular collateral branch of the popliteal artery, the MRI features of which were initially interpreted as suggestive of a soft-tissue sarcoma and which was correctly diagnosed thanks to a systematic pre-biopsy Doppler ultrasound and subsequent CT angiography (CTA).

## Case presentation

An 80-year-old man with a medical history of hypertension and dyslipidemia under medical treatment had presented for nine months with a left popliteal mass that had become progressively bothersome during walking. He was referred to our department after an MRI had been performed elsewhere, with a request for image-guided biopsy of a suspicious popliteal mass. Physical examination revealed a hard, non-pulsatile popliteal swelling that was fixed and non-compressible, adherent to the deeper structures of the popliteal fossa, with no palpable thrill. There was no sign of distal ischemia or local nerve compression. A clinical photograph of the swelling was not obtained at the time of initial presentation.

MRI of the left knee showed a well-circumscribed soft-tissue mass located in the lower third of the thigh on sagittal proton density-weighted imaging, with a markedly heterogeneous signal (Figure 1).

**Figure 1:**
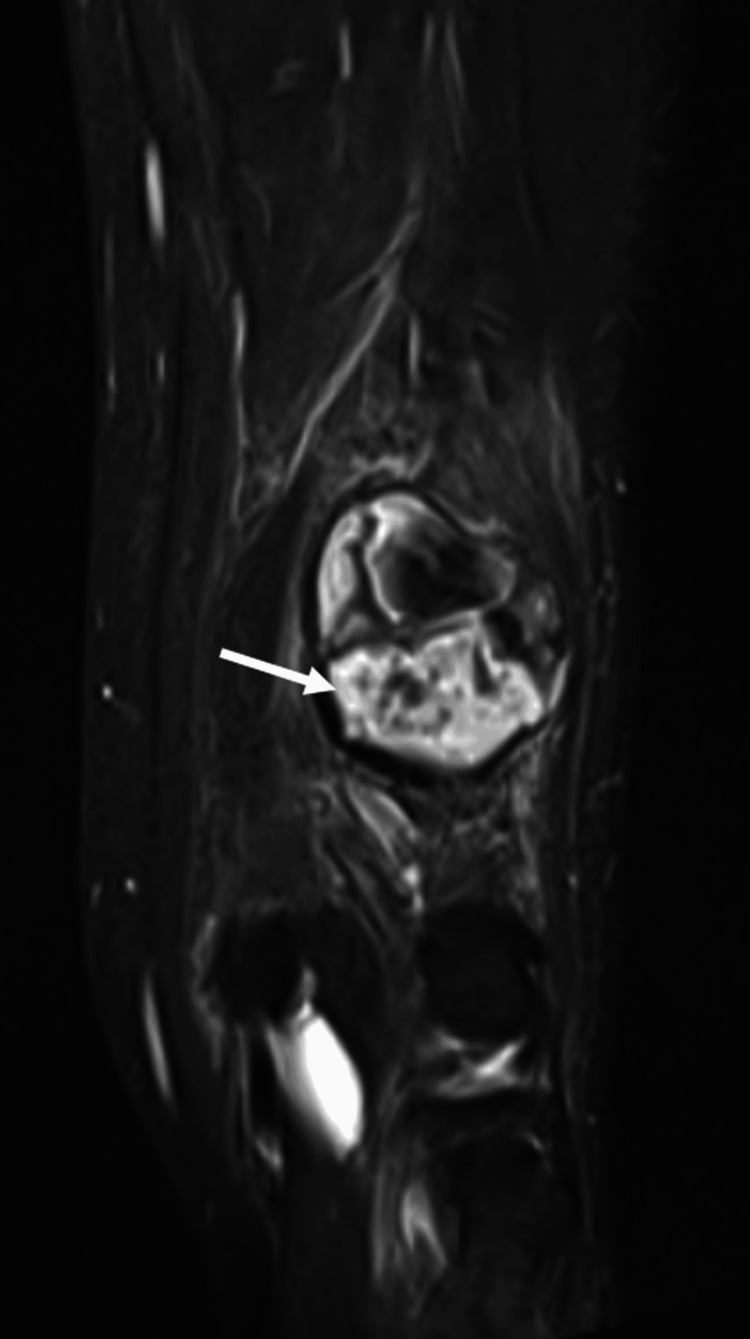
Sagittal proton density-weighted MRI with fat saturation. Heterogeneous soft-tissue mass located in the lower third of the left thigh with well-defined margins. The PD-hyperintense area (arrow) corresponds to thrombus within the aneurysm. PD, proton density

On axial T1-weighted images, hypointense areas within the mass were initially interpreted as a fibrous component, raising the suspicion of a malignant soft-tissue tumor. Although other well-circumscribed soft-tissue masses, such as schwannoma, desmoid tumor, or solitary fibrous tumor, were considered in the differential, the heterogeneous appearance and the hypovascular-appearing fibrous component led to the working hypothesis of a fibrous sarcoma, which prompted the referral for image-guided biopsy (Figure 2).

**Figure 2:**
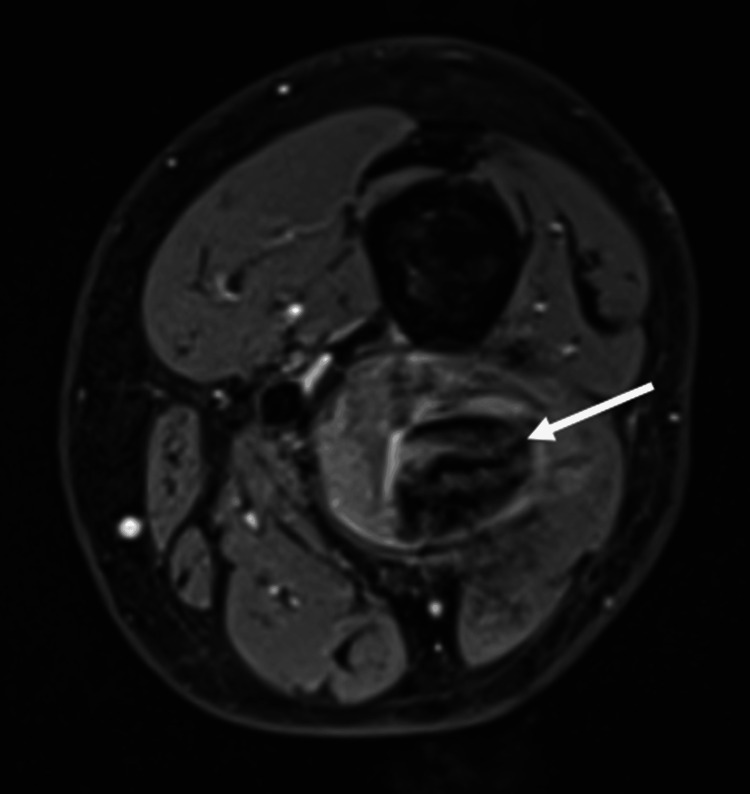
Axial T1-weighted MRI with fat saturation. Heterogeneous soft-tissue lesion. The central hypointense areas (arrow) were initially misinterpreted as a fibrous tumoral component. MRI, magnetic resonance imaging

After gadolinium administration, contrast enhancement was heterogeneous, with markedly hypervascular areas (Figure 3).

**Figure 3:**
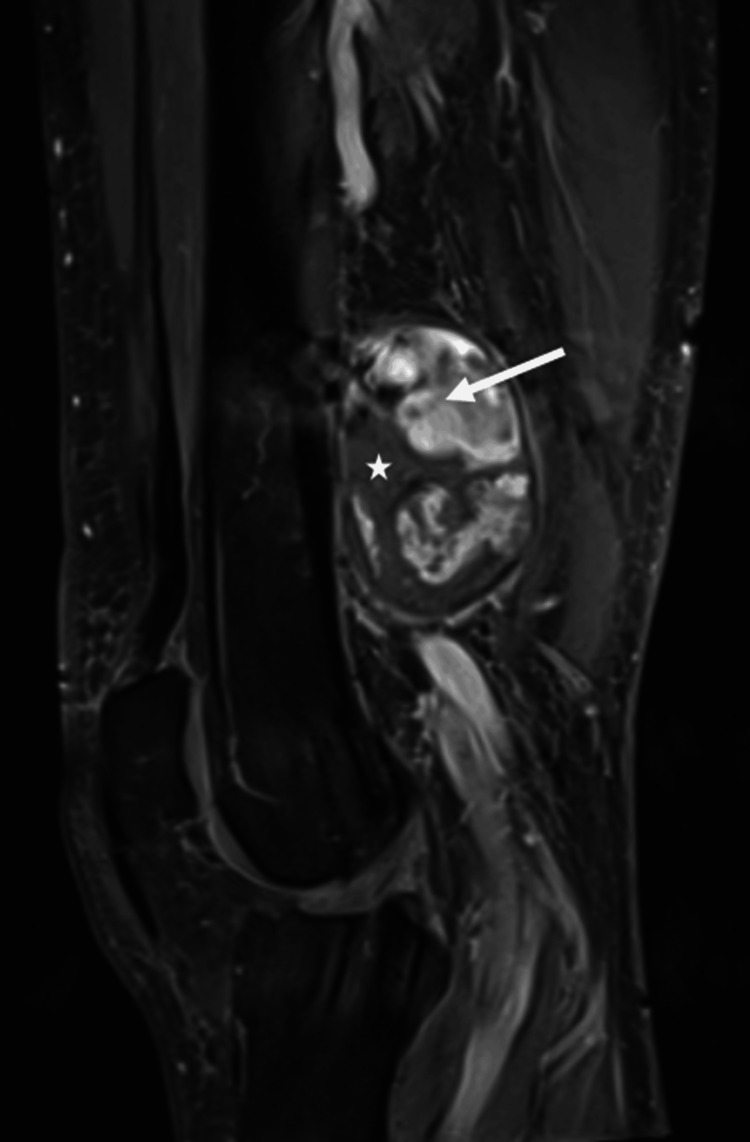
Sagittal contrast-enhanced T1-weighted MRI with fat saturation. Heterogeneous hypervascular appearance of the mass. The hypointense areas on T1 actually correspond to rapid-flow zones (arrow), initially mistaken for a fibrous component. The non-enhancing area (asterisk) corresponds to the thrombus within the aneurysm. MRI, magnetic resonance imaging

The patient had been referred for image-guided biopsy of a suspected soft-tissue mass. As is routine in our institution, a pre-biopsy Doppler ultrasound was performed to assess the vascularity of the lesion before any invasive procedure. It revealed a heterogeneous mass containing intralesional arterial flow, raising the unexpected suspicion of a vascular lesion. Reinterpretation of the MRI in light of the Doppler findings allowed the hypointense areas on T1- and PD-weighted images to be reclassified as organized thrombus and flow void within the aneurysmal sac, rather than as a fibrous tumoral component. As this Doppler ultrasound was not initially intended as a formal diagnostic examination but as a pre-procedural safety check, no images were stored in the PACS at that time. The biopsy was immediately cancelled, and CTA was scheduled for confirmation.

CTA of the lower limbs confirmed the diagnosis, demonstrating a partially thrombosed aneurysm arising from a supra-articular collateral branch of the left popliteal artery, identified as the descending genicular artery, with the feeding vessel clearly individualized, arising from the supra-articular popliteal trunk (Figure 4).

**Figure 4:**
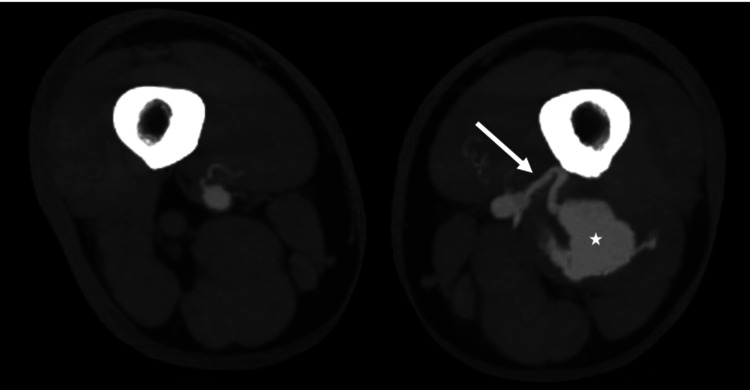
Axial maximum intensity projection (MIP) CT angiography image, arterial phase. Supra-articular branch of the popliteal artery feeding the aneurysmal sac (arrow). The aneurysm is partially thrombosed, with a patent lumen (asterisk).

The aneurysmal sac measured 47 × 60 × 67 mm (anteroposterior × transverse × craniocaudal diameters) and was supplied by the descending genicular artery, arising from the supra-articular segment of the popliteal trunk just above the level of the knee joint. The main popliteal artery itself was of normal caliber, without ectasia or any additional aneurysm. No other popliteal, femoropopliteal, or contralateral lower-limb aneurysm was identified. Three-dimensional volume-rendered reconstructions further facilitated the anatomic analysis (Figure 5).

**Figure 5:**
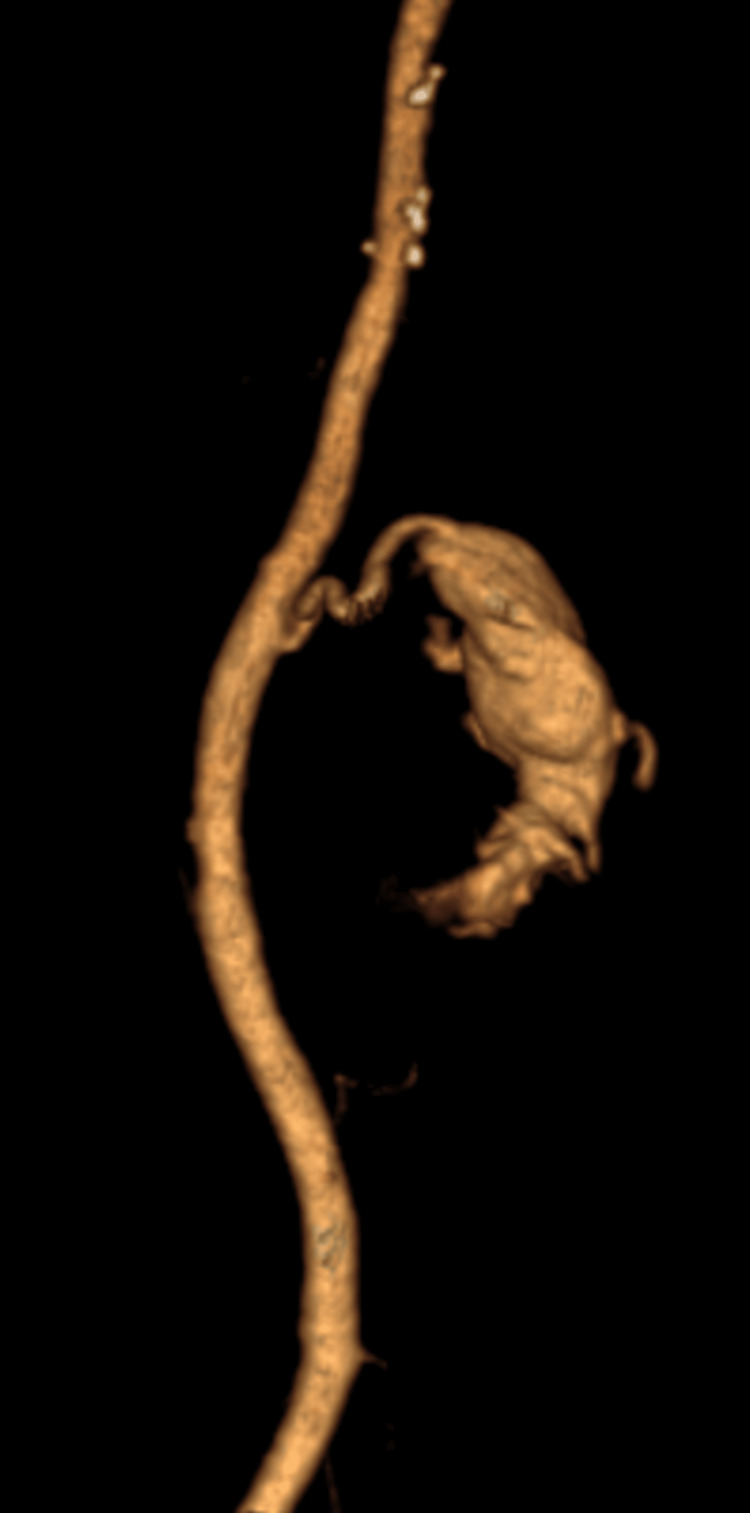
Three-dimensional volume-rendered CT angiography reconstruction. Visualization of the aneurysmal sac and its feeding artery arising from the supra-articular popliteal trunk.

An etiologic workup including inflammatory markers (C-reactive protein and erythrocyte sedimentation rate), complete blood count, ionogram, coagulation profile, and screening for connective tissue diseases (Marfan syndrome, Ehlers-Danlos syndrome), vasculitides, and infectious causes was performed and reported as unremarkable in the patient’s referral file; however, the precise individual laboratory values with reference ranges were not available to the authors at the time of manuscript preparation. In the absence of any traumatic, iatrogenic, or systemic predisposing factor, and given the patient’s advanced age and the diffuse atherosclerotic changes seen on imaging, a degenerative origin with associated atherosclerotic involvement was considered the most likely etiology. The patient was subsequently sent back to his referring physician and underwent surgical management in another hospital, with complete exclusion of both the aneurysm and its feeding artery. The immediate postoperative course was uneventful. Long-term follow-up data (recurrence, limb salvage, and patency of adjacent collateral arteries) are unfortunately not available, as the patient was lost to follow-up after discharge from the operating hospital; this constitutes a limitation of our report.

## Discussion

PAAs are the most common peripheral arterial aneurysms, accounting for approximately 70% of lower-limb aneurysms [1,6]. They occur almost exclusively in men (96%), with a mean age of 65 years. They are bilateral in more than half of cases and are associated with an AAA in approximately 40% of patients; conversely, a PAA is found in approximately 15% of patients with an AAA [1]. Their natural history is characterized by silent growth, with a complication rate reaching 60% to 75% at five years [1]. By contrast, aneurysms developed from the collateral branches of the popliteal artery are exceedingly rare and have been described almost exclusively as case reports, with most of them being post-traumatic or iatrogenic [2,3,4]. True degenerative aneurysms of these branches, as in our patient, are particularly rare and have been only sporadically reported in the literature.

The etiology of PAAs is essentially degenerative, with patients frequently presenting classic cardiovascular risk factors [1]. True degenerative aneurysms must be distinguished from pseudoaneurysms, which result from arterial wall disruption secondary to trauma, iatrogenic injury, or infection [1]. In younger patients, popliteal artery entrapment syndrome (PAES) should always be considered, as repeated mechanical compression by anomalous insertions of the gastrocnemius muscle or by an aberrant arterial course can lead to post-stenotic dilatation and aneurysm formation [7]. Other rarer etiologies include inflammatory or connective tissue diseases such as Marfan syndrome, Ehlers-Danlos syndrome, and vasculitides [1]. In our patient, the advanced age, the absence of muscular insertion abnormalities on imaging, the absence of trauma or iatrogenic context, the unremarkable connective tissue workup, and the diffuse atherosclerotic changes pointed to a degenerative origin, with atherosclerosis identified as a contributing factor rather than the primary cause of the aneurysm.

The clinical presentation of PAAs is variable. Notably, only 44% of PAAs are palpable on clinical examination, which highlights the limited sensitivity of palpation alone [1]. Approximately one-third of patients are asymptomatic at the time of diagnosis [1]. Among these asymptomatic patients, the risk of acute ischemia from thrombosis is estimated between 17% and 46%, with a major amputation risk approaching 50% [1]. Symptomatic forms manifest mainly as acute ischemia from thrombosis or distal embolization, more rarely as chronic ischemia, venous or nerve compression, or rupture (3% of cases) [1]. When extensive mural thrombus is present, the lesion may lose its pulsatile character, as in our patient, making clinical diagnosis even more challenging. In retrospect, our initial clinical evaluation could have been further enriched by systematic auscultation searching for a bruit, which, when audible, may raise early suspicion of a vascular origin and prompt appropriate imaging. We believe that, in any hard, non-pulsatile popliteal mass, systematic auscultation combined with a high index of clinical suspicion for vascular pathology should be considered an essential step before any invasive procedure, even when the initial palpation findings appear to favor a solid tumor.

A partially thrombosed aneurysm can show a markedly heterogeneous signal on imaging: hypointense areas on T1- and T2-weighted images, related either to organized thrombus or to flow void within the aneurysmal lumen, can mimic a fibrous tumoral component and suggest a sarcoma [5]. Albert et al. reported an illustrative case of a profunda femoris artery pseudoaneurysm initially mistaken for a soft-tissue sarcoma, which led to catastrophic hemorrhage during an elective open biopsy [5]. This observation, like ours, highlights that any soft-tissue mass with an unusual appearance must be evaluated for a vascular origin before any invasive procedure [5].

Doppler ultrasound is the first-line investigation for any popliteal mass. Its low cost, non-invasive nature, and wide availability make it ideal for initial evaluation, allowing identification of the circulating component and characterization of mural thrombus [1,5]. In our patient, it was a Doppler ultrasound that prevented an unnecessary and potentially fatal biopsy. CTA is the reference examination for pretherapeutic workup: it precisely depicts the aneurysm location, its relationships with the popliteal trunk and collateral branches, the extent of mural thrombosis, the feeding vessel, and the status of the distal runoff [1]. When MRI is performed first, the presence of intralesional flow void or close anatomic relationships with an arterial trajectory should alert the radiologist to a possible vascular origin and prompt MR angiography or complementary Doppler ultrasound before any invasive procedure [5].

This case also underscores the value of systematic pre-biopsy Doppler ultrasound as a routine safety check before any percutaneous procedure on an unexplained soft-tissue mass. Even when intended only as a quick assessment of lesional vascularity, this simple, rapid, and inexpensive step can detect unsuspected vascular lesions and prevent catastrophic hemorrhagic complications [5]. We believe such a pre-procedural ultrasound check should be encouraged as a standard of care before image-guided biopsies of any deep soft-tissue mass.

The complete diagnostic and therapeutic journey of our patient, from initial symptom onset to surgical exclusion, is summarized in Figure 6.

**Figure 6:**
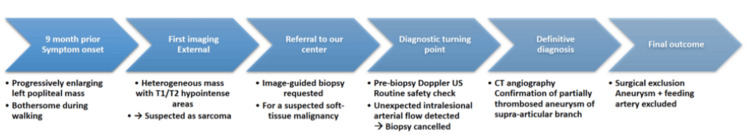
Clinical timeline of the patient's diagnostic and therapeutic journey. Figure created by the authors using Microsoft PowerPoint (no generative AI was used).

The mechanism by which a partially thrombosed aneurysm can mimic a soft-tissue sarcoma on MRI is illustrated schematically in Figure 7. On non-enhanced MRI, the same hypointense T1- and PD-weighted signal can result from two completely different physical phenomena: organized collagen fibers (or necrotic component) in a fibrous tumor, or organized mural thrombus combined with intraluminal flow void in a partially thrombosed aneurysm. Post-contrast imaging is the key differentiator: the aneurysm shows early vascular-type peripheral enhancement of its patent lumen, whereas the fibrous sarcoma shows late, heterogeneous enhancement of its fibrous matrix.

**Figure 7:**
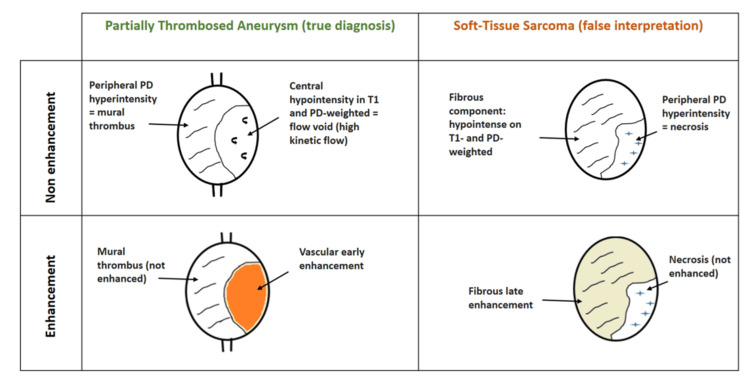
Schematic illustration of the MRI vascular pitfall: soft-tissue sarcoma versus partially thrombosed aneurysm. Figure created by authors using Microsoft PowerPoint (no generative AI was used). MRI, magnetic resonance imaging; PD, proton density

The 2022 Society for Vascular Surgery (SVS) clinical practice guidelines recommend interventional treatment for any symptomatic PAA or for asymptomatic PAAs with a diameter of 2 cm or more, mural thrombus, or distal runoff impairment [8]. Below these thresholds, clinical and Doppler ultrasound surveillance every six months is acceptable [1]. Open surgical exclusion with venous bypass using the great saphenous vein remains the gold standard. It yields excellent results in asymptomatic PAAs, with five-year patency rates above 90% to 95% and limb salvage rates of 95% to 100%; these figures decrease to 70%-80% and 80%, respectively, in symptomatic cases, and to 59% in critical limb ischemia [1,9]. Endovascular treatment with covered self-expanding stent grafts (e.g., Gore Viabahn) is a valid alternative in patients at high surgical risk or with favorable anatomy (supra-articular location, angulation <60°, healthy proximal and distal landing zones > 1 cm), with reported five-year patency rates of approximately 85% and limb salvage rates of 97%, although thrombosis (7%-25%), endoleak (10%-20%), and stent fracture (17%) remain a concern [1].

For aneurysms of the collateral branches of the popliteal artery, the treatment is surgical and relies on complete exclusion of both the aneurysm and its feeding artery. Sacrifice of the feeding branch does not expose to a significant risk of distal ischemia, because the rich anastomotic network of the adjacent collateral arteries ensures effective recruitment and maintains adequate perfusion downstream. In our patient, this strategy was successfully applied with surgical exclusion of the aneurysm and its feeding artery, with an uneventful postoperative course.

## Conclusions

Aneurysms arising from collateral branches of the popliteal artery are rare and may present as a soft-tissue mass with a misleading imaging appearance. When partially thrombosed, their MRI features can mimic a sarcoma and lead to a diagnostic error. Doppler ultrasound, which is fast, inexpensive, and widely available, should be performed before any biopsy of a popliteal mass to rule out a vascular origin. CTA further refines the diagnosis and guides therapeutic management. The key message of this case is straightforward: never biopsy a popliteal mass without first excluding a vascular cause.
